# Association of blood cadmium with all-cause and cause-specific mortality in patients with hypertension

**DOI:** 10.3389/fpubh.2023.1106732

**Published:** 2023-07-04

**Authors:** Shuaijie Chen, Ruming Shen, Jiayi Shen, Lingchun Lyu, Tiemin Wei

**Affiliations:** ^1^Department of Cardiology, Lishui Hospital, College of Medicine, Zhejiang University, Lishui, China; ^2^College of Medicine, Zhejiang University, Hangzhou, China

**Keywords:** blood cadmium, mortality, cardiovascular risk, hypertension, NHANES

## Abstract

**Background:**

Cadmium is a commonly found heavy metal with a prolonged biological half-life, which results in long-term health burden for the population. Prior studies have demonstrated an association between blood cadmium and hypertension. However, few studies examined the relationship between blood cadmium and long-term health outcomes in patients with hypertension. This study aimed to investigate the association of blood cadmium with mortality in patients with hypertension.

**Methods:**

This study analyzed data from the National Health and Nutrition Examination Survey 1999–2012. Complex sampling-weighted multivariate Cox proportional hazards models were used to evaluate the hazard ratios (HRs) of all-cause, cardiovascular, and Alzheimer’s disease mortality in patients with hypertension classified by blood cadmium concentrations’ quantiles.

**Results:**

The study included 12,208 patients with hypertension with a median follow-up duration of 10.8 years. During this period, there were 4,485 all-cause deaths, including 1,520 cardiovascular deaths and 180 Alzheimer’s disease deaths. Compared with the lowest quintile of blood cadmium (≤0.25 μg/L) group, the highest quintile of blood cadmium (≥0.80 μg/L) group’s adjusted HRs were 1.85 (95% CI, 1.59–2.14) for all-cause mortality, 1.76 (95% CI, 1.33–2.34) for cardiovascular mortality, and 3.41 (95% CI, 1.54–7.51) for Alzheimer’s disease mortality. Additionally, the adjusted HR for cardiovascular mortality was 2.12 (95% CI, 1.36–3.30) in never-smoking patients with hypertension.

**Conclusion:**

Higher blood cadmium is associated with increased risks of all-cause, cardiovascular, and Alzheimer’s disease mortality in patients with hypertension. The effect of blood cadmium on cardiovascular mortality may be more pronounced in never-smoking hypertensive patients.

## Introduction

Cadmium is a common toxic heavy metal that is widely present in the environment. It has an extremely long biological half-life in both the environment (approximately 10–30 year) and the human body (greater than 20 years), leading to a long-term health burden (1–3). The exposure of cadmium mainly occurs through industrial production (e.g., mining, metallurgical industries, and manufacturers of nickel-Cd batteries) (4), tobacco smoking, ambient air (5), and foods (6). Although exposure levels have decreased in recent years, cadmium still has a significant impact on various health status, including cardiovascular diseases, diabetes, cognitive dysfunction, Alzheimer’s disease, kidney injury, arthritis, and cancer (3, 7–12). Since the 1950s, multiple studies have suggested an association between cadmium and blood pressure and hypertension (13–17). A recent meta-analysis reconfirmed the positive association between blood cadmium and hypertension (18). In addition, some studies have shown the effect of cadmium exposure on mortality (19–26). Previous studies mostly focus on the general population (20, 22–24). And several studies have focused on cadmium-contaminated areas (25, 26). However, few studies have investigated the association between blood cadmium and long-term health outcomes in high-risk populations who had already suffered from related diseases (e.g., patients with hypertension). There is an urgent need to generate more scientific evidence on the relationship between cadmium exposure and mortality in high-risk populations to raise public awareness of cadmium exposure reduction. Therefore, this study aimed to evaluate the association of blood cadmium with all-cause and cause-specific mortality in patients with hypertension, which may provide reference for the long-term adverse health effects of cadmium exposure, especially in high-risk population.

## Methods

### Study participants

The National Health and Nutrition Examination Survey (NHANES) is a program designed to assess the health and nutritional status of the US population (27). The US National Center for Health Statistics (NCHS) is responsible for producing vital and health statistics by using a stratified, multi-stage probability sampling design that ensures participants are representative of the civilian deinstitutionalized population of the United States (27). The NHANES protocol has been approved by the NCHS Research Ethics Review Board, and written informed consent has been obtained from each participant (28).

**Figure 1:**
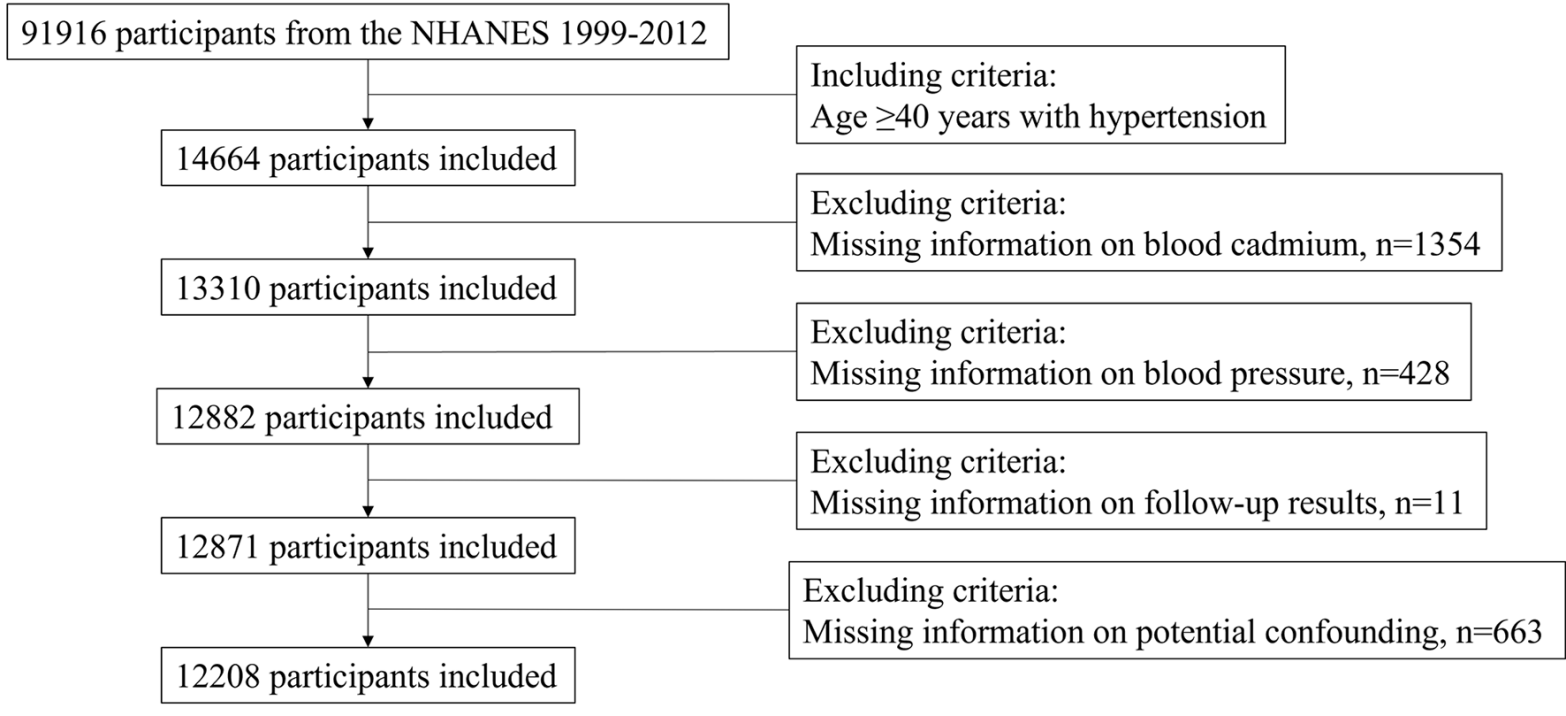
Flowchart of participants selection.

For this study, we obtained data on NHANES participants between 1999 and 2012. Since there were few mortality-related cases under the age of 40, we focused on participants aged 40 years or older with hypertension at baseline, resulting in a total of 14,664 participants. Hypertension was defined as measured systolic blood pressure (SBP) ≥ 140 mmHg or/and diastolic blood pressure (DBP) ≥ 90 mmHg, or/and previous diagnosis of hypertension, or/and taking antihypertensive prescription. We excluded patients with missing information on blood cadmium (*n* = 1,354), missing blood pressure data (*n* = 428), lacking follow-up data (*n* = 11), and missing information on potential confounding variables (*n* = 663). This left us with a final sample size of 12,208 participants for analysis (Figure 1).

### Measurement of blood cadmium

Whole blood specimens are processed, stored, and shipped to the Division of Laboratory Sciences, National Center for Environmental Health, and Centers for Disease Control and Prevention for analysis. Blood cadmium was determined using a PerkinElmer Model SIMAA 6000 simultaneous multi-element atomic absorption spectrometer with Zeeman background correction in NHANES 1999–2002 and using inductively coupled plasma mass spectrometry in NHANES 2003–2012 (27).

The extremely high and low values were verified by NCHS staff carefully, and numerous consistency checks were performed. The limit of detection (LOD) for blood cadmium was 0.1–0.2 μg/L. Blood cadmium below LOD was replaced with a value equal to LOD divided by the square root of two. Blood cadmium below LOD were found in 12% of participants overall (*n* = 1,488), which did not affect grouping according to quintile.

### Outcome ascertainment

The NHANES linked mortality data used in this study were updated with follow-up data through December 31, 2019 (29). All-cause death was defined as death from any cause. According to the International Classification of Diseases, 10th revision (ICD-10), cardiovascular death was defined as death due to diseases of heart (I00-I09, I11, I13, and I20–I51) and cerebrovascular diseases (I60–I69) (30). Alzheimer’s disease death was defined as death due to Alzheimer’s disease (G30).

### Covariate assessment

The following variables were obtained through the interview questionnaire: age, sex, race/ethnicity, educational level, smoking status, medical conditions (including hypertension, diabetes, asthma, heart failure, coronary heart disease, stroke, emphysema, chronic bronchitis, liver condition, and cancer), and antihypertensive medication use. The following variables were measured according to standard protocols: body mass index (BMI; calculated as weight in kilograms divided by height in meters squared), blood pressure, total cholesterol, triglycerides, high density lipoprotein, serum creatinine, uric acid, and cotinine. The estimated glomerular filtration rate (eGFR) was calculated by the Chronic Kidney Disease Epidemiology Collaboration equation (31). Obese was defined as BMI ≥ 30 kg/m^2^. Optimal blood pressure was defined as SBP < 140 mmHg and DBP < 90 mmHg.

### Statistical analysis

Given that the NHANES data used in this study were not simple random samples, appropriate weights were used for each analysis based on the selected variables, as recommended by the US Centers for Disease Control and Prevention (32). Continuous variables were expressed as mean ± standard error (mean ± SE), and comparisons between groups were made by analysis of variance. Categorical variables were expressed as percentages, and comparisons between groups were made by the chi-square test. Complex sampling-weighted multivariate Cox models were applied to investigate the hazard ratio (HR) and 95% confidence interval (CI) between blood cadmium and mortality. Model 1 was adjusted for age, sex, race/ethnicity, and education level. Model 2 was further adjusted for BMI, eGFR, SBP, DBP, total cholesterol, triglycerides, high-density lipoprotein, and disease conditions (heart failure, coronary heart disease, stroke, diabetes, and cancer). Model 3 was further adjusted for serum cotinine and smoking status (never, former, or current smoker). Based on model 3, subgroup analyses were stratified by gender, age, BMI, blood pressure control, eGFR, and smoking status. Interactions between blood cadmium (lowest and highest quintiles) and stratification factors were assessed. Restricted cubic spline regression was used to explore the dose–response association of blood cadmium and mortality. Furthermore, we performed sensitivity analysis by using the random forest imputation method to interpolate values of blood cadmium below LOD and missing variables. All statistical analyses were performed using R version 4.1.2 (R Project for Statistical Computing). *p* < 0.05 was regarded as statistically significant for all tests.

## Results

### Baseline characteristics

In this study, a total of 12,208 patients with hypertension aged 40 years or older were included. During a median follow-up of 10.8 years (interquartile range [IQR], 7.9–14.4), 4,485 all-cause deaths occurred, including 1,520 cardiovascular deaths and 180 Alzheimer’s disease deaths. The range and percentage of blood cadmium below the limit of detection were 0.1–0.2 μg/L and 12.2%, respectively. The baseline characteristics of the patients grouped according to blood cadmium quintiles were listed in Table 1. Participants with higher blood cadmium levels were more likely to be older, female, non-Hispanic black, non-obese, and current smokers. They also had lower education levels, lower triglycerides, lower eGFR, lower DBP, and higher SBP, higher total cholesterol, and higher high-density lipoprotein levels. In addition, participants with higher blood cadmium levels had a lower prevalence of diabetes but a higher prevalence of cardiovascular diseases (heart failure, coronary heart disease, and stroke).

**Table 1:** Participants baseline demographic and clinical characteristics.

Characteristic	Total	Blood cadmium (μg/L)					*p* value	*p* value for trend
		Quintile 1 (≤0.25)	Quintile 2 (0.26–0.38)	Quintile 3 (0.39–0.5)	Quintile 4 (0.51–0.79)	Quintile 5 (≥0.8)		
Age, years	60.70 ± 0.18	56.76 ± 0.28	60.62 ± 0.28	63.22 ± 0.34	64.29 ± 0.39	59.89 ± 0.33	<0.001	<0.001
Gender, %								
Male	47.4	60.4	47.3	40.6	39.2	46	<0.001	<0.001
Female	52.6	39.6	52.7	59.4	60.8	54	<0.001	<0.001
Race, %								
Mexican American	4.6	5	4.8	4.9	4.6	3.2	0.001	0.003
Other hispanic	3.9	3.9	4.6	5.1	3.1	2.6	0.004	0.03
Non-hispanic white	75.3	76.5	76.2	75.9	76.9	70.6	<0.001	0.006
Non-hispanic black	11.8	11.5	10.9	9.9	11	15.7	<0.001	<0.001
Other race	4.5	3	3.5	4.2	4.3	8	<0.001	<0.001
Education level, %								
Less than high school	22.8	14.8	19.2	23.8	25	33.7	<0.001	<0.001
At least high school	77.2	85.2	80.8	76.2	75	66.3	<0.001	<0.001
Smoking status, %								
Current	17.1	1.2	2.1	5.7	18.1	65.6	<0.001	<0.001
Former	34.8	29.9	36.5	42.5	46.1	21.3	<0.001	0.08
Never	48	69	61.4	51.8	35.8	13.2	<0.001	<0.001
Cotinine, ng/ml	51.90 ± 1.68	12.07 ± 2.05	13.59 ± 1.70	17.95 ± 1.76	51.00 ± 3.37	182.80 ± 4.22	<0.001	<0.001
Body mass index, kg/m2	30.00 ± 0.09	31.75 ± 0.19	30.63 ± 0.18	29.77 ± 0.16	29.28 ± 0.17	28.00 ± 0.15	<0.001	<0.001
Body mass index, %								
<30	56.9	47.1	52.5	58.7	61.1	68.2	<0.001	<0.001
≥30	43.1	52.9	47.5	41.3	38.9	31.8	<0.001	<0.001
Systolic blood pressure, mmHg	136.71 ± 0.28	133.56 ± 0.43	135.24 ± 0.50	139.49 ± 0.51	138.06 ± 0.61	137.97 ± 0.66	<0.001	<0.001
Diastolic blood pressure, mmHg	73.70 ± 0.27	75.21 ± 0.41	73.99 ± 0.39	73.15 ± 0.44	72.15 ± 0.57	73.38 ± 0.52	<0.001	<0.001
Total Cholesterol, mmol/L	5.27 ± 0.02	5.19 ± 0.03	5.23 ± 0.03	5.33 ± 0.03	5.31 ± 0.03	5.31 ± 0.04	0.015	0.005
Triglycerides, mmol/L	1.94 ± 0.02	2.07 ± 0.05	1.97 ± 0.04	1.85 ± 0.04	1.85 ± 0.04	1.92 ± 0.04	0.003	<0.001
High density lipoprotein, mmol/L	1.37 ± 0.43	1.28 ± 0.38	1.36 ± 0.40	1.40 ± 0.43	1.44 ± 0.45	1.39 ± 0.48	<0.001	<0.001
Creatinine, umol/L	83.69 ± 0.52	82.71 ± 0.54	82.96 ± 0.78	81.47 ± 1.18	85.28 ± 1.27	86.89 ± 1.69	0.033	0.002
eGFR,ml/min/1.73m^2^	81.94 ± 0.34	85.26 ± 0.50	81.11 ± 0.56	80.64 ± 0.64	78.28 ± 0.70	83.19 ± 0.67	<0.001	<0.001
eGFR, %								
<60	15.4	10	14.4	16.7	20.9	16.9	<0.001	<0.001
≥60	84.6	90	85.6	83.3	79.1	83.1	<0.001	<0.001
Diabetes, %	16.1	20.1	16.4	14.4	14.7	13.7	<0.001	<0.001
Heart failure, %	5.2	3.3	4.3	5.1	7.4	7	<0.001	<0.001
Coronary heart disease, %	7.6	5.7	6.9	8.6	8.8	8.8	0.001	<0.001
Stroke, %	5.8	3.1	5.7	5.4	7.5	8.2	<0.001	<0.001
Cancer, %	15	13.3	13.8	16.3	18.5	13.9	<0.001	0.045

eGFR, estimated glomerular filtration rate.

Continuous variables are expressed as means and standard error and categorical variables as percentages. Means and percentages are weighted.

Linear trends across categories of blood cadmium quintiles were assessed with linear regression for continuous variables and chi-square tests for categorical variables.

### Association of blood cadmium with mortality

Table 2 presented survey-weighted Cox regression results. The multivariate adjusted model 3 showed that blood cadmium was positively associated with all-cause, cardiovascular, and Alzheimer’s disease mortality in participants. Compared with participants in the lowest quintile (≤0.25 μg/L) of blood cadmium, participants in the highest quintile (≥0.8 μg/L) had HRs of 1.85 (95% CI, 1.59–2.14) for all-cause mortality, 1.76 (95% CI, 1.33–2.34) for cardiovascular mortality, and 3.41 (95% CI, 1.54–7.51) for Alzheimer’s disease mortality. Moreover, the effect of blood cadmium on mortality may be in a dose-dependent manner.

**Table 2:** Hazard ratios for all-cause, cardiovascular and Alzheimer’s disease mortality of all participants, stratified by blood cadmium.

Outcomes	Blood cadmium (μg/L)					*p* value for trend
	Quintile 1 (≤0.25)	Quintile 2 (0.26–0.38)	Quintile 3 (0.39–0.5)	Quintile 4 (0.51–0.79)	Quintile 5 (≥0.8)	
All-cause mortality						
Unadjusted HR	1 [Ref]	**1.41(1.22–1.63)**	**1.85(1.59–2.15)**	**2.55(2.22–2.93)**	**2.86(2.48–3.30)**	**<0.001**
*p* value		**<0.001**	**<0.001**	**<0.001**	**<0.001**	
Model 1 HR	1 [Ref]	1.10(0.95–1.28)	**1.19(1.04–1.35)**	**1.44(1.26–1.65)**	**2.37(2.07–2.70)**	**<0.001**
*p* value		0.192	**0.009**	**<0.001**	**<0.001**	
Model 2 HR	1 [Ref]	1.12(0.97–1.30)	**1.25(1.11–1.41)**	**1.50(1.31–1.72)**	**2.41(2.11–2.75)**	**<0.001**
*p* value		0.119	**<0.001**	**<0.001**	**<0.001**	
Model 3 HR	1 [Ref]	1.09(0.94–1.26)	**1.18(1.05–1.34)**	**1.33(1.16–1.53)**	**1.85(1.59–2.14)**	**<0.001**
*p* value		0.259	**0.006**	**<0.001**	**<0.001**	
Cardiovascular mortality						
Unadjusted HR	1 [Ref]	**1.47(1.10–1.98)**	**1.93(1.50–2.49)**	**2.48(1.92–3.21)**	**2.52(1.96–3.25)**	**<0.001**
*p* value		**0.01**	**<0.001**	**<0.001**	**<0.001**	
Model 1 HR	1 [Ref]	1.11(0.82–1.49)	1.15(0.92–1.45)	1.27(0.99–1.64)	**1.97(1.55–2.52)**	**<0.001**
*p* value		0.507	0.224	0.064	**<0.001**	
Model 2 HR	1 [Ref]	1.11(0.83–1.50)	1.23(0.98–1.53)	**1.35(1.05–1.73)**	**2.06(1.61–2.64)**	**<0.001**
*p* value		0.473	0.071	**0.019**	**<0.001**	
Model 3 HR	1 [Ref]	1.09(0.81–1.48)	1.19(0.96–1.49)	1.26(0.97–1.63)	**1.76(1.33–2.34)**	**<0.001**
*p* value		0.556	0.119	0.083	**<0.001**	
Alzheimer’s disease mortality						
Unadjusted HR	1 [Ref]	**2.94(1.40–6.19)**	**4.51(2.39–8.50)**	**5.10(2.67–9.74)**	**4.12(2.21–7.70)**	**<0.001**
*p* value		**0.005**	**<0.001**	**<0.001**	**<0.001**	
Model 1 HR	1 [Ref]	2.02(0.96–4.24)	**2.20(1.14–4.25)**	**1.92(0.98–3.77)**	**2.98(1.50–5.92)**	**0.014**
*p* value		0.124	**0.026**	**0.077**	**0.003**	
Model 2 HR	1 [Ref]	1.99(0.94–4.19)	**2.11(1.07–4.13)**	1.83(0.94–3.55)	**2.86(1.43–5.74)**	**0.021**
*p* value		0.071	**0.03**	0.075	**0.003**	
Model 3 HR	1 [Ref]	2.02(0.96–4.27)	**2.18(1.10–4.29)**	**1.98(1.00–3.90)**	**3.41(1.54–7.51)**	**0.014**
*p* value		0.066	**0.025**	**0.049**	**0.002**	

HR, hazard ratio; Ref, reference; eGFR, estimated glomerular filtration rate; SBP, systolic blood pressure; and DBP, diastolic blood pressure.

Model 1: adjusted for age, sex, race/ethnicity, and education level.

Model 2: adjustments for model 1 plus BMI, eGFR, SBP, DBP, total cholesterol, triglycerides, high-density lipoprotein, and disease conditions (heart failure, coronary heart disease, stroke, diabetes, and cancer).

Model 3: adjustments for model 2 plus serum cotinine and smoking status (never, former, or current smoker).

*p* value for trend was obtained from Cox proportional hazards models with blood cadmium quintiles as a continuous variable.

Statistically significant HR and p-values were shown in bold.

### Dose–response curves for blood cadmium and mortality

**Figure 2:**
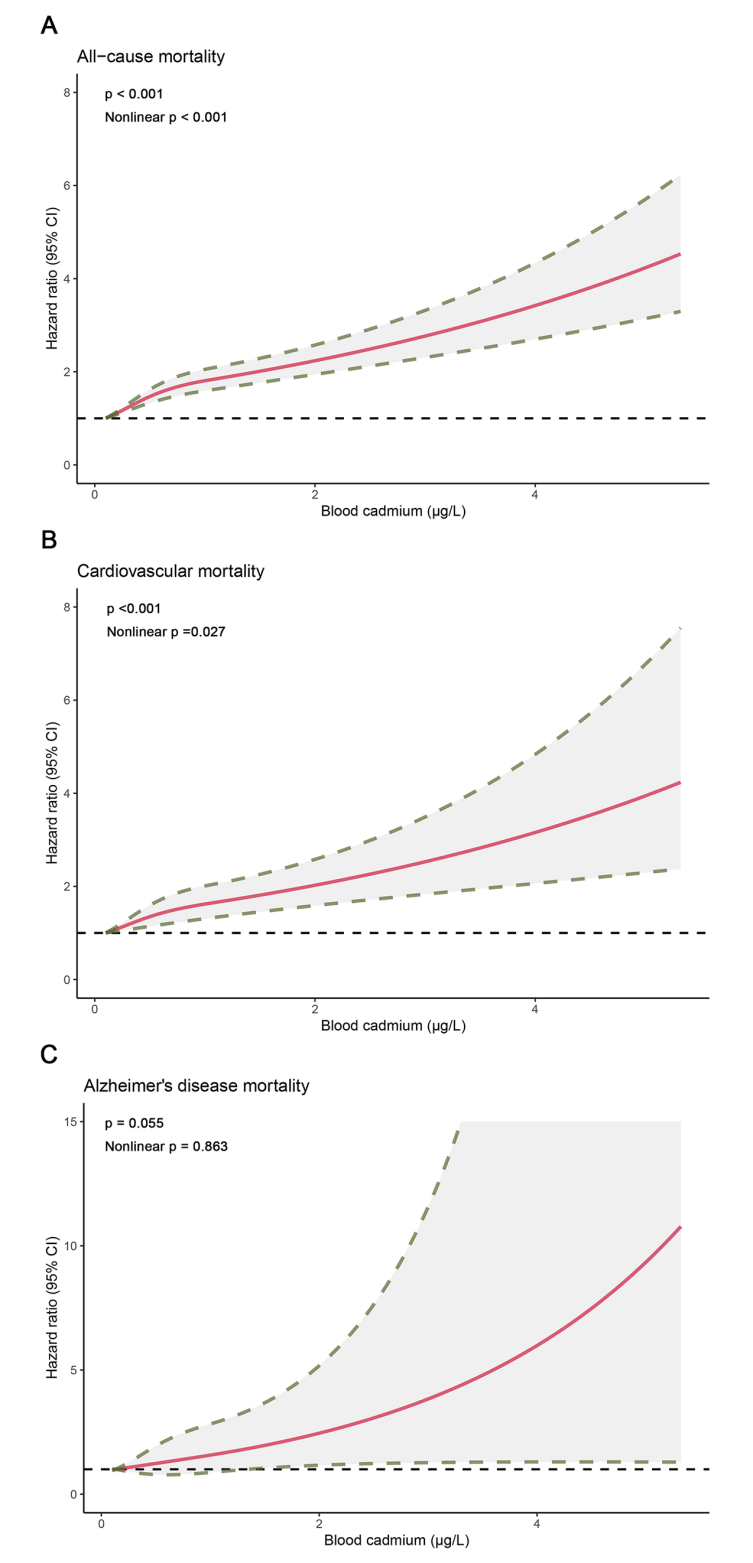
Dose–response curves for concentrations of blood cadmium and mortality. **(A)** All-cause mortality. **(B)** Cardiovascular mortality. **(C)** Alzheimer’s disease mortality.

The restricted cubic spline analysis showed a dose–response association between blood cadmium and all-cause, cardiovascular, and Alzheimer’s disease mortality (Figure 2), which was consistent with the weighted Cox regression results. We observed a non-linear association for all-cause (*p* < 0.001, Nonlinear *p* < 0.001) and cardiovascular mortality (*p* < 0.001, Nonlinear *p* = 0.027), with adjusted HRs steeper at lower blood cadmium concentrations than at higher concentrations. For Alzheimer’s disease mortality, the association was close to a linear correlation (*p* = 0.055, Nonlinear *p* = 0.863).

### Subgroup analysis

**Figure 3:**
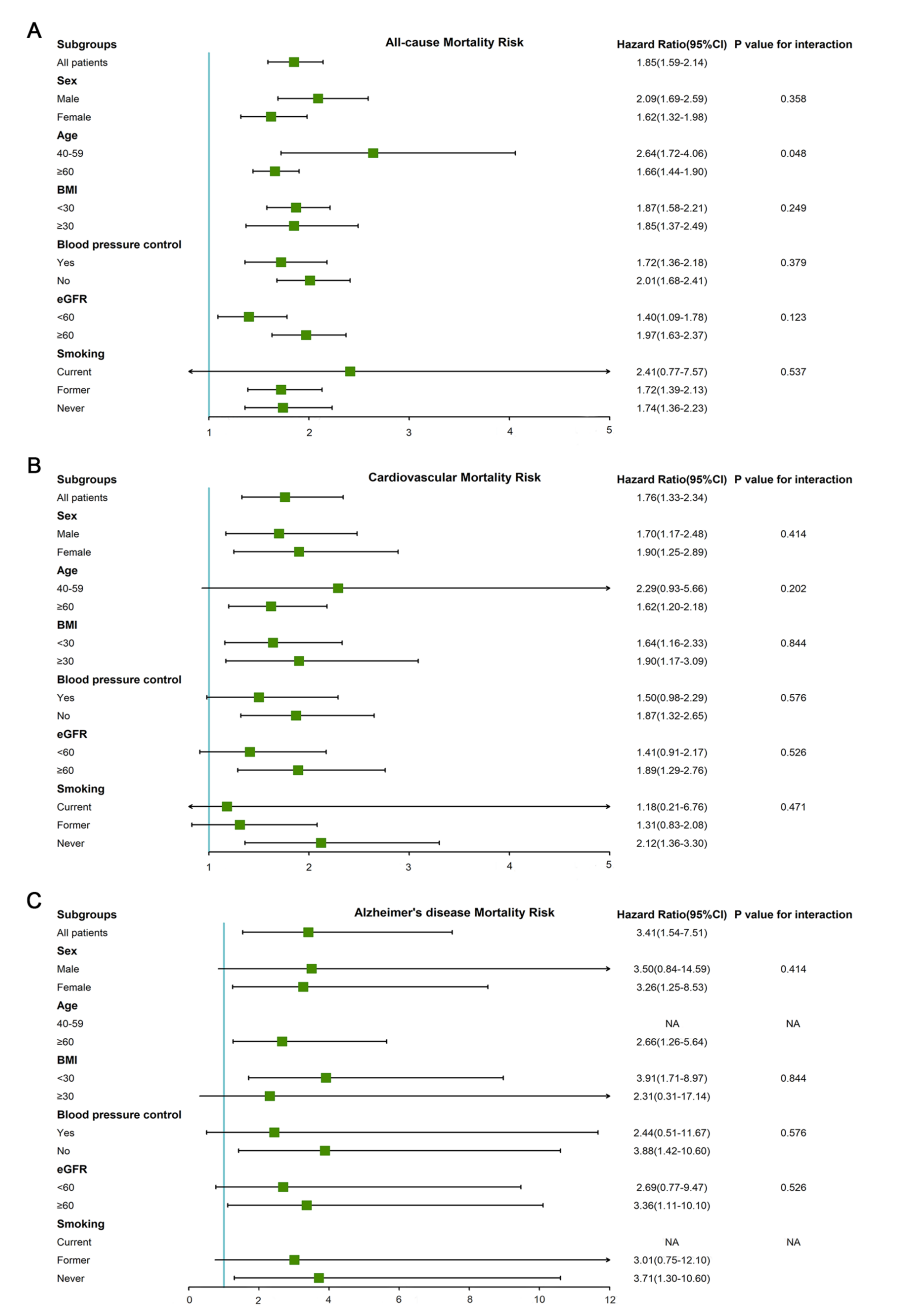
The adjusted HRs of the highest quintile of blood cadmium compared with the lowest quintile of blood cadmium in subgroups (gender, age, BMI, blood pressure control, eGFR, and smoking status). **(A)** All-cause mortality. **(B)** Cardiovascular mortality. **(C)** Alzheimer’s disease mortality.

The results of subgroup analysis according to multivariate adjusted model 3 showed that the significant association between blood cadmium and mortality still existed in most subgroups (Supplementary Table 1). The adjusted HRs of the highest quintile of blood cadmium compared with the lowest quintile of blood cadmium and interactions in subgroups were shown in Figure 3. For all-cause mortality, there was a significant interaction between age and blood cadmium (*p* = 0.048). The HR of the highest blood cadmium quintile compared to the lowest blood cadmium quintile in middle-aged (40–59 years) participants was 2.64 (95% CI, 1.72–4.06), while the HR of older participants (≥60 years) was only 1.66 (95% CI, 1.44–1.90). For cardiovascular mortality, there was no statistical significance in the Cox regression results of the blood cadmium quintiles of the current smokers (the highest quintile of blood cadmium, HR 1.18 [95% CI, 0.21–6.76]) or former smokers (the highest quintile of blood cadmium, HR 1.31 [95% CI, 0.83–2.08]). But blood cadmium was significantly associated with an increased risk of cardiovascular mortality in never-smoking participants (the highest quintile of blood cadmium, HR 2.12 [95% CI, 1.36–3.30]). For Alzheimer’s disease mortality, the HR of the highest blood cadmium quintile was higher in non-obese participants (HR 3.91 [95% CI, 1.71–8.97]) compared to obese participants (HR 2.31 [95% CI, 0.31–17.14]).

### Association of blood cadmium with mortality in sensitivity analysis

As shown in Supplementary Figure 1, triglycerides exhibited the highest proportion of missing data (11.26%, *n* = 1,650). Therefore, we conducted imputation for blood cadmium below LOD and missing variables. Supplementary Table 2 presented a similar positive association of blood cadmium with all-cause, cardiovascular and Alzheimer’s disease mortality. Compared to participants in the lowest quintile (≤0.25 μg/L) of blood cadmium, those in the highest quintile (≥0.8 μg/L) had the HRs of 1.76 (95%CI, 1.54–2.01) for all-cause mortality, 1.82 (95%CI, 1.44–2.31) for cardiovascular mortality and 2.57 (95%CI, 1.33–4.98) for Alzheimer’s disease mortality. Supplementary Figure 2 demonstrated the significant dose–response association of blood cadmium with all-cause and cardiovascular mortality after the interpolation.

## Discussion

Our study suggests that blood cadmium levels are linked to higher risks of all-cause, cardiovascular, and Alzheimer’s mortality in patients with hypertension, even after adjusting for smoking status and cotinine concentrations. Moreover, a positive dose–response curve was observed between blood cadmium and mortality. Notably, in patients with low blood cadmium levels, the risks associated with elevated blood cadmium were even more pronounced. Furthermore, the effect of blood cadmium on cardiovascular mortality was more significant in never-smoking patients with hypertension than in smoking patients with hypertension.

Many previous studies have investigated the association of blood cadmium and mortality. Nawrot et al. (33) found that blood cadmium was associated with all-cause and non-cardiovascular mortality in a population with relatively high cadmium exposure. Several NHANES studies have suggested that blood cadmium is associated with all-cause, cardiovascular, and cancer mortality in the US general population (19, 34, 35). Similar results were also observed among patients with type 2 diabetes (36). Moreover, a few studies have suggested that higher blood cadmium is associated with higher mortality from influenza and pneumonia, as well as Alzheimer’s disease (37, 38). Our study also found a positive correlation between blood cadmium and mortality in a hypertensive population. Studies among the US general population suggest that the effect of blood cadmium in increasing the risk of cardiovascular death is more significant in smokers than in non-smokers (19, 34). However, our study among a hypertensive population found that the effect of blood cadmium in increasing the risk of cardiovascular death was more pronounced in participants who never smoked. This may be related to the different effects of blood cadmium on blood pressure in patients with different smoking status. Based on the 1999–2004 NHANES, Tellez-Plaza et al. proposed that blood cadmium is associated with increased blood pressure, and this association is mainly reflected in never-smokers.

Additionally, the dose–response curves in our study showed that the increased risk of cardiovascular mortality was more pronounced with the increase in blood cadmium at lower levels of exposure. Furthermore, the blood cadmium level of never-smokers was significantly lower than that of smokers. This finding suggests that the cardiovascular mortality risk of never-smoking patients with hypertension is more susceptible to the influence of blood cadmium than smoking patients with hypertension. However, more precisely designed prospective studies are needed to confirm this finding. It is recommended that patients with hypertension should reduce cadmium exposure as much as possible, and even non-smokers may benefit from reducing cadmium exposure from various dietary and environmental sources.

Our results also showed that higher blood cadmium had lower prevalence of diabetes, which are contrary to previous study (8). Possible explanations are as follows: Urban residents have a higher burden of cadmium pollution compared to rural residents, but they have a lower incidence of diabetes than rural residents (39, 40). The mean value±SE of blood cadmium in our study was 0.57 ± 0.01ug/L, which was much lower than the blood cadmium biological threshold limit value for non-occupational exposure recommended by the World Health Organization.^1^ The cadmium exposure in our study may not be high enough to induce overt diabetes (41).

Blood cadmium has been linked to increased risk of cardiovascular mortality, possibly due to its effects on blood pressure and atherosclerosis. Studies have suggested that cadmium may cause oxidative stress (42), activate calcium channels (43), inhibit nitric oxide (44), and vascular tissue damage by leading to endothelial dysfunction (45) Additionally, there is evidence to suggest that cadmium-induced oxidative stress may lead to renal injury, which in turn indirectly affects the cardiovascular system (10). Moreover, cadmium may play a role in the formation of amyloid plaques and neuronal damage, potentially contributing to Alzheimer’s disease mortality risk. Given that hypertension is a risk factor for Alzheimer’s disease, blood cadmium may indirectly affect Alzheimer’s disease through its impact on blood pressure. However, the mechanism behind the increased mortality risk associated with blood cadmium exposure requires further investigation. It is recommended that further studies be conducted to clarify the precise mechanisms involved and identify effective preventive measures.

Although this is the first study to investigate the effect of blood cadmium on the mortality of patients with hypertension, we acknowledge several limitations. Blood cadmium was measured only once, which may not accurately reflect long-term blood cadmium levels. Moreover, the low proportion of blood cadmium below the detection limit still affects the accuracy of the dose–response curve. Additionally, outcome variables identified by death certificates may not fully reflect the exact cause of death. Finally, this study is an observational study, and despite correction for multiple confounding variables, there is still a risk of confounding bias. Nonetheless, this study has several strengths, including a large sample size and a long follow-up time. The Cox model fully adjusted for many traditional risk factors, as well as smoking status and cotinine concentration.

## Conclusion

Our study found that higher blood cadmium levels are associated with an increased risk of all-cause, cardiovascular, and Alzheimer’s disease mortality in patients with hypertension. The association remained significant after adjusting for demographic characteristics, serum cotinine, smoking status, BMI, eGFR, blood pressure, lipids, and various disease conditions. Notably, the effect of blood cadmium on cardiovascular mortality may be more pronounced in never-smoking patients with hypertension than in smoking patients with hypertension. Considering the risk of poor long-term health outcomes, cadmium exposure is recommended to be reduced as much as possible in patients with hypertension, even non-smokers.

## Data availability statement

Publicly available datasets were analyzed in this study. This data can be found here: The NHANES website: NHANES Questionnaires, Datasets, and Related Documentation (https://wwwn.cdc.gov/nchs/nhanes/Default.aspx).

## Ethics statement

The studies involving human participants were reviewed and approved by The NCHS Research Ethics Review Board approved the NHANES protocol (https://www.cdc.gov/nchs/nhanes/irba98.htm). The patients/participants provided their written informed consent to participate in this study.

## Author contributions

TW, LL, and SC conceived and designed research. RS and JS processed data and performed statistical analysis. SC and RS wrote the initial paper. TW and LL reviewed and corrected the article. All authors contributed to the article and approved the submitted version.

## Funding

This work was supported by Science and Technology Public Welfare Project of Lishui city (2019GYX28) and Key Research and Development Program of Zhejiang Province (2020ZJZC01).

## Conflict of interest

The authors declare that the research was conducted in the absence of any commercial or financial relationships that could be construed as a potential conflict of interest.

## Publisher’s note

All claims expressed in this article are solely those of the authors and do not necessarily represent those of their affiliated organizations, or those of the publisher, the editors and the reviewers. Any product that may be evaluated in this article, or claim that may be made by its manufacturer, is not guaranteed or endorsed by the publisher.

## Footnotes

^1^http://www.inchem.org/documents/ehc/ehc/ehc134.htm

## Abbreviations

HR: Hazard ratio
CI: Confidence interval
NHANES: National Health and Nutrition Examination Survey
NCHS: National Center for Health Statistics
SBP: Systolic blood pressure
DBP: Diastolic blood pressure
ICD-10: International Classification of Diseases, 10th revision
BMI: Body mass index
eGFR: Estimated glomerular filtration rate
SE: Standard error
Ref: Reference
