# Supplementary material for: Association of blood cadmium with all-cause and cause-specific mortality in patients with hypertension
Source: Front Public Health. 2023 Jul 4;11:1106732. doi: 10.3389/fpubh.2023.1106732 (PMC10353433; doi:10.3389/fpubh.2023.1106732)
Supplement: Supplementary file 1 [file Table_3.DOCX]

**Supplementary Table 1. Multivariate model 3 hazard ratios for mortality of** **subgroups (gender, age, BMI, blood pressure control, eGFR and smoking status), stratified by blood cadmium.**

| **Outcomes** | **deaths/n** | **Blood cadmium (μg/L)** | | | | | **P value for trend** |
| --- | --- | --- | --- | --- | --- | --- | --- |
|  |  | **Quintile 1 (≤0.25)** | **Quintile 2 (0.26–0.38)** | **Quintile 3 (0.39–0.5)** | **Quintile 4 (0.51–0.79)** | **Quintile 5 (≥0.8)** |  |
| **All-cause mortality** | |  |  |  |  |  |  |
| Sex |  |  |  |  |  |  |  |
| Male | 2386/6013 | 1 [Ref] | 1.04(0.85-1.28) | **1.27(1.07-1.50)** | **1.48(1.21-1.80)** | **2.09(1.69-2.59)** | **<0.001** |
| P value |  |  | 0.697 | **0.006** | **<0.001** | **<0.001** |  |
| Female | 2099/6195 | 1 [Ref] | 1.13(0.92-1.40) | 1.10(0.94-1.30) | 1.20(0.98-1.46) | **1.62(1.32-1.98)** | **<0.001** |
| P value |  |  | 0.241 | 0.243 | 0.074 | **<0.001** |  |
| Age |  |  |  |  |  |  |  |
| 40-59 | 579/4341 | 1 [Ref] | 1.02(0.69-1.50) | 1.28(0.86-1.89) | **1.88(1.23-2.86)** | **2.64(1.72-4.06)** | **<0.001** |
| P value |  |  | 0.94 | 0.22 | **0.003** | **<0.001** |  |
| ≥60 | 3906/7867 | 1 [Ref] | 1.10(0.95-1.27) | **1.15(1.03-1.28)** | **1.22(1.07-1.39)** | **1.66(1.44-1.90)** | **<0.001** |
| P value |  |  | 0.204 | **0.014** | **0.003** | **<0.001** |  |
| BMI |  |  |  |  |  |  |  |
| <30 | 2913/7041 | 1 [Ref] | 1.11(0.94-1.31) | **1.24(1.08-1.42)** | **1.39(1.19-1.63)** | **1.87(1.58-2.21)** | **<0.001** |
| P value |  |  | 0.228 | **0.002** | **<0.001** | **<0.001** |  |
| ≥30 | 1572/5167 | 1 [Ref] | 1.09(0.86-1.39) | 1.15(0.93-1.42) | **1.24(0.98-1.58)** | **1.85(1.37-2.49)** | **<0.001** |
| P value |  |  | 0.456 | 0.188 | **0.047** | **<0.001** |  |
| Blood pressure control | |  |  |  |  |  |  |
| Yes | 1981/6302 | 1 [Ref] | 0.96(0.79-1.17) | 1.04(0.86-1.26) | **1.39(1.12-1.72)** | **1.72(1.36-2.18)** | **<0.001** |
| P value |  |  | 0.705 | 0.688 | **0.003** | **<0.001** |  |
| No | 2504/5906 | 1 [Ref] | **1.24(1.03-1.50)** | **1.34(1.15-1.57)** | **1.31(1.09-1.56)** | **2.01(1.68-2.41)** | **<0.001** |
| P value |  |  | **0.022** | **<0.001** | **0.003** | **<0.001** |  |
| eGFR |  |  |  |  |  |  |  |
| <60 | 1460/2268 | 1 [Ref] | 1.04(0.81-1.33) | 1.18(0.97-1.44) | 1.14(0.90-1.46) | **1.40(1.09-1.78)** | **0.009** |
| P value |  |  | 0.787 | 0.099 | 0.281 | **0.007** |  |
| ≥60 | 3025/9940 | 1 [Ref] | 1.09(0.90-1.32) | 1.13(0.95-1.33) | **1.35(1.14-1.60)** | **1.97(1.63-2.37)** | **<0.001** |
| P value |  |  | 0.355 | 0.167 | **<0.001** | **<0.001** |  |
| Smoking |  |  |  |  |  |  |  |
| Current | 786/2063 | 1 [Ref] | 0.40(0.09-1.67) | 1.97(0.62-6.31) | 1.83(0.57-5.86) | 2.41(0.77-7.57) | **0.003** |
| P value |  |  | 0.207 | 0.252 | 0.306 | 0.133 |  |
| Former | 1828/4224 | 1 [Ref] | 1.00(0.78-1.28) | 1.15(0.93-1.42) | **1.36(1.08-1.70)** | **1.72(1.39-2.13)** | **<0.001** |
| P value |  |  | 0.989 | 0.195 | **0.009** | **<0.001** |  |
| Never | 1871/5921 | 1 [Ref] | 1.14(0.94-1.38) | 1.10(0.93-1.31) | 1.16(0.98-1.38) | **1.74(1.36-2.23)** | **<0.001** |
| P value |  |  | 0.196 | 0.28 | 0.09 | **<0.001** |  |
| **Cardiovascular mortality** | |  |  |  |  |  |  |
| Sex |  |  |  |  |  |  |  |
| Male | 816/6013 | 1 [Ref] | 0.99(0.68-1.44) | 1.19(0.89-1.59) | 1.26(0.90-1.78) | **1.70(1.17-2.48)** | **0.003** |
| P value |  |  | 0.953 | 0.239 | 0.182 | **0.006** |  |
| Female | 704/6195 | 1 [Ref] | 1.30(0.89-1.91) | 1.28(0.93-1.76) | 1.31(0.90-1.91) | **1.90(1.25-2.89)** | **0.011** |
| P value |  |  | 0.179 | 0.129 | 0.156 | **0.003** |  |
| Age |  |  |  |  |  |  |  |
| 40-59 | 157/4341 | 1 [Ref] | 0.98(0.41-2.34) | 1.17(0.61-2.25) | 1.10(0.51-2.35) | 2.29(0.93-5.66) | 0.087 |
| P value |  |  | 0.967 | 0.63 | 0.815 | 0.073 |  |
| ≥60 | 1363/7867 | 1 [Ref] | 1.11(0.81-1.52) | 1.17(0.95-1.45) | 1.22(0.94-1.59) | **1.62(1.20-2.18)** | **0.002** |
| P value |  |  | 0.515 | 0.135 | 0.141 | **0.002** |  |
| BMI |  |  |  |  |  |  |  |
| <30 | 959/7041 | 1 [Ref] | 1.07(0.72-1.58) | 1.23(0.91-1.67) | 1.24(0.90-1.70) | **1.64(1.16-2.33)** | **0.002** |
| P value |  |  | 0.739 | 0.182 | 0.191 | **0.005** |  |
| ≥30 | 561/5167 | 1 [Ref] | 1.17(0.78-1.76) | 1.20(0.82-1.76) | 1.26(0.83-1.93) | **1.90(1.17-3.09)** | **0.018** |
| P value |  |  | 0.447 | 0.352 | 0.274 | **0.01** |  |
| Blood pressure control | |  |  |  |  |  |  |
| Yes | 631/6302 | 1 [Ref] | 1.00(0.65-1.53) | 1.12(0.78-1.61) | 1.38(0.96-1.99) | 1.50(0.98-2.29) | **0.003** |
| P value |  |  | 0.982 | 0.55 | 0.087 | 0.062 |  |
| No | 889/5906 | 1 [Ref] | 1.21(0.83-1.75) | 1.24(0.92-1.68) | 1.14(0.82-1.59) | **1.87(1.32-2.65)** | **0.001** |
| P value |  |  | 0.326 | 0.161 | 0.441 | **<0.001** |  |
| eGFR |  |  |  |  |  |  |  |
| <60 | 539/2268 | 1 [Ref] | 0.99(0.65-1.49) | 1.06(0.76-1.49) | 1.06(0.70-1.62) | 1.41(0.91-2.17) | 0.107 |
| P value |  |  | 0.947 | 0.729 | 0.782 | 0.123 |  |
| ≥60 | 981/9940 | 1 [Ref] | 1.16(0.81-1.68) | 1.20(0.90-1.60) | 1.31(0.96-1.80) | **1.89(1.29-2.76)** | **<0.001** |
| P value |  |  | 0.417 | 0.205 | 0.089 | **<0.001** |  |
| Smoking |  |  |  |  |  |  |  |
| Current | 250/2063 | 1 [Ref] | 0.44(0.06-3.42) | 1.11(0.17-7.32) | 0.95(0.17-5.44) | 1.18(0.21-6.76) | 0.395 |
| P value |  |  | 0.432 | 0.915 | 0.954 | 0.85 |  |
| Former | 591/4224 | 1 [Ref] | 0.80(0.49-1.29) | 1.11(0.75-1.64) | 1.09(0.71-1.68) | 1.31(0.83-2.08) | 0.084 |
| P value |  |  | 0.353 | 0.614 | 0.697 | 0.248 |  |
| Never | 679/5921 | 1 [Ref] | 1.31(0.91-1.89) | 1.13(0.84-1.50) | 1.27(0.89-1.81) | **2.12(1.36-3.30)** | **0.024** |
| P value |  |  | 0.151 | 0.423 | 0.181 | **<0.001** |  |
| **Alzheimer's disease mortality** | |  |  |  |  |  |  |
| Sex |  |  |  |  |  |  |  |
| Male | 80/6013 | 1 [Ref] | 1.84(0.62-5.47) | **2.82(1.10-7.27)** | 2.41(0.87-6.69) | 3.50(0.84-14.59) | 0.083 |
| P value |  |  | 0.273 | **0.032** | 0.091 | 0.085 |  |
| Female | 100/6195 | 1 [Ref] | 2.16(0.79-5.93) | 1.72(0.72-4.11) | 1.54(0.61-3.87) | **3.26(1.25-8.53)** | 0.069 |
| P value |  |  | 0.135 | 0.219 | 0.358 | **0.016** |  |
| Age |  |  |  |  |  |  |  |
| 40-59 | 4/4341 | NA | NA | NA | NA | NA | NA |
| P value |  |  |  |  |  |  |  |
| ≥60 | 176/7867 | 1 [Ref] | 1.94(0.91-4.13) | **2.00(1.01-3.98)** | 1.86(0.95-3.66) | **2.66(1.26-5.64)** | **0.027** |
| P value |  |  | 0.087 | **0.047** | 0.07 | **0.011** |  |
| BMI |  |  |  |  |  |  |  |
| <30 | 129/7041 | 1 [Ref] | 1.85(0.78-4.38) | 2.13(0.95-4.76) | 2.10(0.97-4.54) | **3.91(1.71-8.97)** | **0.003** |
| P value |  |  | 0.164 | 0.066 | 0.061 | **0.001** |  |
| ≥30 | 51/5167 | 1 [Ref] | 2.45(0.62-9.71) | 1.91(0.59-6.26) | 1.52(0.39-5.91) | 2.31(0.31-17.14) | 0.719 |
| P value |  |  | 0.201 | 0.283 | 0.546 | 0.412 |  |
| Blood pressure control | |  |  |  |  |  |  |
| Yes | 76/6302 | 1 [Ref] | **3.54(1.21-10.39)** | **3.60(1.37-9.43)** | **3.09(1.14-8.32)** | 2.44(0.51-11.67) | 0.431 |
| P value |  |  | **0.021** | **0.009** | **0.026** | 0.263 |  |
| No | 104/5906 | 1 [Ref] | 1.15(0.44-2.99) | 1.47(0.62-3.52) | 1.42(0.55-3.65) | **3.88(1.42-10.60)** | **0.008** |
| P value |  |  | 0.781 | 0.383 | 0.47 | **0.008** |  |
| eGFR |  |  |  |  |  |  |  |
| <60 | 59/2268 | 1 [Ref] | 1.41(0.38-5.19) | 1.40(0.36-5.39) | 1.72(0.48-6.23) | 2.69(0.77-9.47) | 0.067 |
| P value |  |  | 0.608 | 0.625 | 0.406 | 0.123 |  |
| ≥60 | 121/9940 | 1 [Ref] | 2.21(0.91-5.40) | **2.45(1.12-5.34)** | 2.08(0.90-4.83) | **3.36(1.11-10.10)** | 0.077 |
| P value |  |  | 0.08 | **0.024** | 0.087 | **0.031** |  |
| Smoking |  |  |  |  |  |  |  |
| Current | 9/2063 | NA | NA | NA | NA | NA | NA |
| P value |  |  |  |  |  |  |  |
| Former | 68/4224 | 1 [Ref] | 1.22(0.30-4.93) | 2.65(0.73-9.71) | 2.17(0.65-7.20) | 3.01(0.75-12.10) | 0.113 |
| P value |  |  | 0.776 | 0.14 | 0.207 | 0.121 |  |
| Never | 103/5921 | 1 [Ref] | 2.41(0.98-5.95) | 1.90(0.83-4.37) | 1.55(0.65-3.71) | **3.71(1.30-10.60)** | 0.13 |
| P value |  |  | 0.056 | 0.13 | 0.328 | **0.015** |  |
| HR, Hazard ratio; Ref, reference; eGFR, estimated glomerular filtration rate; SBP, systolic blood pressure; DBP, diastolic blood pressure.  Model 3: adjustments for age, sex, race/ethnicity, education level, BMI, eGFR, SBP, DBP, total cholesterol, triglycerides, high-density lipoprotein, disease conditions (heart failure, coronary heart disease, stroke, diabetes, and cancer), serum cotinine and smoking status (never, former, or current smoker).  P value for trend was obtained from Cox proportional hazards models with blood cadmium quintiles as a continuous variable.  Statistically significant HR and p-values were shown in bold. | | | | | | | |
