# Supplementary material for: Association of blood cadmium with all-cause and cause-specific mortality in patients with hypertension
Source: Front Public Health. 2023 Jul 4;11:1106732. doi: 10.3389/fpubh.2023.1106732 (PMC10353433; doi:10.3389/fpubh.2023.1106732)
Supplement: Supplementary file 2 [file Table_4.DOCX]

**Supplementary Table 2. Hazard ratios for all-cause, cardiovascular and** **Alzheimer's disease mortality of all participants** **after the interpolation, stratified by blood cadmium.**

| **Outcomes** | **Blood cadmium (μg/L)** | | | | | **P value for trend** |
| --- | --- | --- | --- | --- | --- | --- |
|  | **Quintile 1 (≤0.25)** | **Quintile 2 (0.26–0.38)** | **Quintile 3 (0.39–0.5)** | **Quintile 4 (0.51–0.79)** | **Quintile 5 (≥0.8)** |  |
| **All-cause mortality** | | | | | | |
| Unadjusted HR | 1 [Ref] | **1.32 [1.15, 1.52]** | **1.70 [1.47, 1.97]** | **2.30 [2.02, 2.62]** | **2.76 [2.43, 3.13]** | **<0.001** |
| P value |  | **<0.001** | **<0.001** | **<0.001** | **<0.001** |  |
| Model 1 HR | 1 [Ref] | 1.01 [0.88, 1.16] | 1.09 [0.96, 1.24] | **1.32 [1.18, 1.48]** | **2.21 [1.96, 2.49]** | **<0.001** |
| P value |  | 0.891 | 0.207 | **<0.001** | **<0.001** |  |
| Model 2 HR | 1 [Ref] | 1.03 [0.90, 1.17] | 1.13 [1.00, 1.27] | **1.39 [1.25, 1.55]** | **2.26 [2.01, 2.54]** | **<0.001** |
| P value |  | 0.703 | 0.056 | **<0.001** | **<0.001** |  |
| Model 3 HR | 1 [Ref] | 0.99 [0.87, 1.13] | 1.07 [0.95, 1.21] | **1.26 [1.13, 1.41]** | **1.76 [1.54, 2.01]** | **<0.001** |
| P value |  | 0.899 | 0.272 | **<0.001** | **<0.001** |  |
| **Cardiovascular mortality** | | | | | | |
| Unadjusted HR | 1 [Ref] | **1.45 [1.13, 1.87]** | **1.97 [1.57, 2.48]** | **2.36 [1.90, 2.95]** | **2.64 [2.11, 3.28]** | **<0.001** |
| P value |  | **0.003** | **<0.001** | **<0.001** | **<0.001** |  |
| Model 1 HR | 1 [Ref] | 1.06 [0.83, 1.35] | 1.17 [0.95, 1.45] | **1.23 [1.01, 1.51]** | **1.99 [1.61, 2.44]** | **<0.001** |
| P value |  | 0.632 | 0.145 | **0.042** | **<0.001** |  |
| Model 2 HR | 1 [Ref] | 1.07 [0.84, 1.37] | 1.21 [0.99, 1.49] | **1.32 [1.08, 1.61]** | **2.10 [1.71, 2.60]** | **<0.001** |
| P value |  | 0.587 | 0.064 | **0.007** | **<0.001** |  |
| Model 3 HR | 1 [Ref] | 1.05 [0.82, 1.35] | 1.18 [0.97, 1.45] | **1.25 [1.03, 1.53]** | **1.82 [1.44, 2.31]** | **<0.001** |
| P value |  | 0.688 | 0.102 | **0.026** | **<0.001** |  |
| **Alzheimer's disease mortality** | | | | | | |
| Unadjusted HR | 1 [Ref] | **2.82 [1.51, 5.24]** | **3.43 [1.87, 6.28]** | **4.26 [2.59, 7.02]** | **3.51 [2.08, 5.92]** | **<0.001** |
| P value |  | **0.001** | **<0.001** | **<0.001** | **<0.001** |  |
| Model 1 HR | 1 [Ref] | **1.82 [1.00, 3.31]** | 1.66 [0.91, 3.03] | **1.67 [1.02, 2.75]** | **2.34 [1.34, 4.09]** | 0.052 |
| P value |  | **0.048** | 0.098 | **0.042** | **0.003** |  |
| Model 2 HR | 1 [Ref] | 1.81 [0.99, 3.29] | 1.62 [0.88, 3.00] | 1.62 [0.98, 2.67] | **2.21 [1.24, 3.94]** | 0.085 |
| P value |  | 0.052 | 0.122 | 0.062 | **0.007** |  |
| Model 3 HR | 1 [Ref] | **1.84 [1.01, 3.35]** | 1.67 [0.89, 3.11] | **1.71 [1.03, 2.83]** | **2.57 [1.33, 4.98]** | 0.055 |
| P value |  | **0.045** | 0.107 | **0.038** | **0.005** |  |
| HR, Hazard ratio; Ref, reference; eGFR, estimated glomerular filtration rate; SBP, systolic blood pressure; DBP, diastolic blood pressure.  Model 1: adjusted for age, sex, race/ethnicity, and education level.  Model 2: adjustments for model 1 plus BMI, eGFR, SBP, DBP, total cholesterol, triglycerides, high-density lipoprotein, and disease conditions (heart failure, coronary heart disease, stroke, diabetes, and cancer).  Model 3: adjustments for model 2 plus serum cotinine and smoking status (never, former, or current smoker).  P value for trend was obtained from Cox proportional hazards models with blood cadmium quintiles as a continuous variable.  Statistically significant HR and p-values were shown in bold. | | | | | | |
