# Supplementary figures and images for: Association of blood cadmium with all-cause and cause-specific mortality in patients with hypertension
Source: Front Public Health. 2023 Jul 4;11:1106732. doi: 10.3389/fpubh.2023.1106732 (PMC10353433; doi:10.3389/fpubh.2023.1106732)

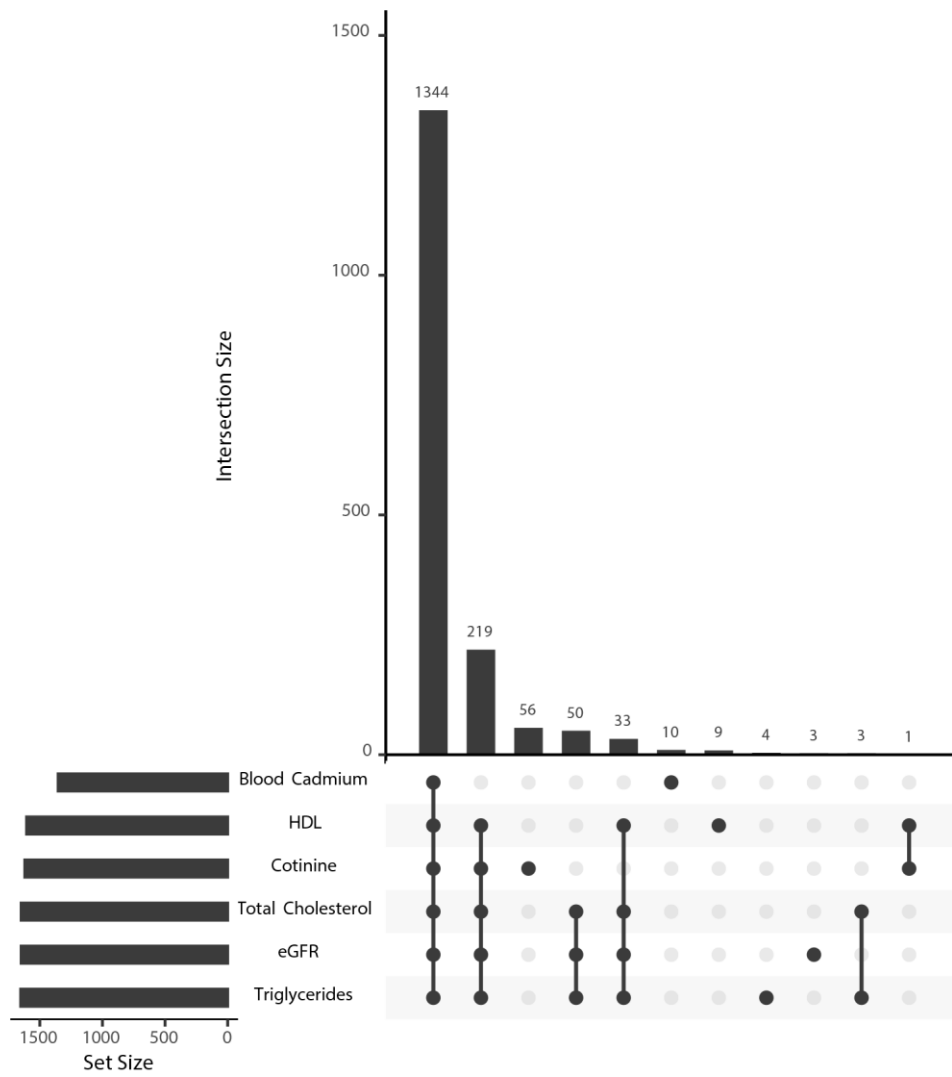

**Supplementary Figure 1. Interpolation diagram of missing variables.**

Supplement: Supplementary file 3 [file Image_1.pdf]
