# Supplementary material for: Association of blood cadmium with all-cause and cause-specific mortality in patients with hypertension
Source: Front Public Health. 2023 Jul 4;11:1106732. doi: 10.3389/fpubh.2023.1106732 (PMC10353433; doi:10.3389/fpubh.2023.1106732)
Supplement: Supplementary file 4 [file Image_2.pdf]

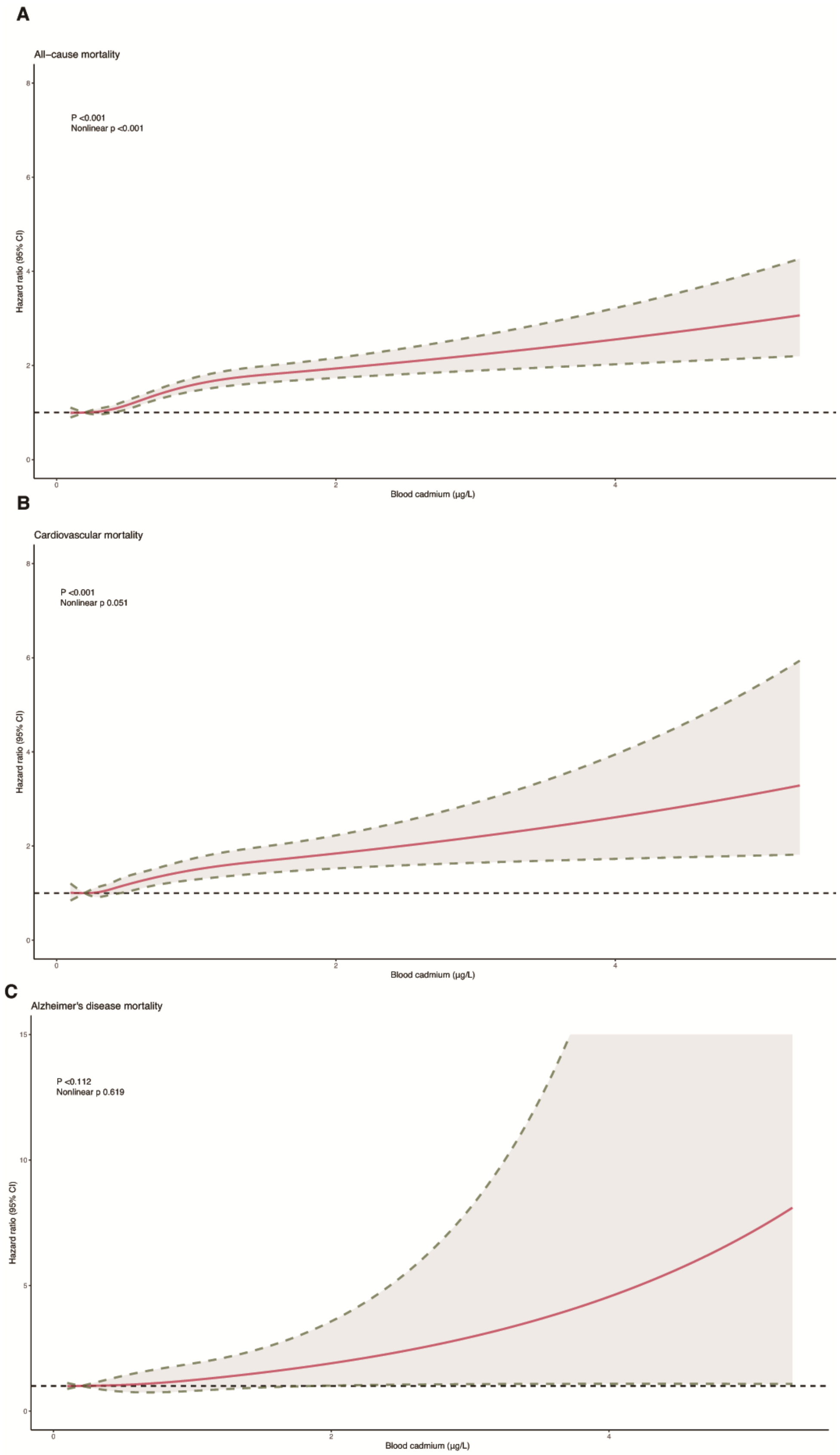

Supplementary Figure 2. Dose-response curves for concentrations of blood cadmium and mortality after the interpolation. (A) All-cause mortality. (B) Cardiovascular mortality. (C) Alzheimer's disease mortality.
